# Left Pulvinar Thalamic Tumor with Ventricular Atrial Extension Presenting as Network-Level Cognitive and Gait Dysfunction

**DOI:** 10.3390/diagnostics16060836

**Published:** 2026-03-11

**Authors:** Florin Mihail Filipoiu, Stefan Oprea, Cosmin Pantu, Matei Șerban, Răzvan-Adrian Covache-Busuioc, Corneliu Toader, Mugurel Petrinel Radoi, Octavian Munteanu, Raluca Florentina Tulin

**Affiliations:** 1 Faculty of General Medicine, “Carol Davila” University of Medicine and Pharmacy, 050474 Bucharest, Romania; florin.filipoiu@umfcd.com (F.M.F.); mateiserban@innbn.com (M.Ș.);; 2 Department of Anatomy, “Carol Davila” University of Medicine and Pharmacy, 050474 Bucharest, Romania; 3 Department of Neurosurgery, “Carol Davila” University of Medicine and Pharmacy, 050474 Bucharest, Romania; 4 Department of Vascular Neurosurgery, National Institute of Neurology and Neurovascular Diseases, 077160 Bucharest, Romania; 5 Puls Med Association, 051885 Bucharest, Romania

**Keywords:** thalamic tumor, intraventricular extension, ventricular atrium, thalamocortical networks, executive dysfunction, aphasia, dual-task gait, intracranial compliance, physiology-guided microsurgery, network phenotyping

## Abstract

**Background and Clinical Significance:** Deep thalamic and periventricular lesions are uncommon in adults but can result in significant loss of function because of their convergence on three interdependent processes: thalamocortical state regulation, throughput of periventricular long association systems, and ventricular compartmental compliance. The resulting combination of executive control collapse, retrieval-weighted language fragility, and load-sensitive gait instability may occur early after a lesion forms an atrial/posterior horn interface, and pressure-linked autonomic symptoms may be late to develop. Screening deficits will likely be minimal and therefore underreported. **Objective/Aim:** To present a thalamic–atrial/posterior horn tumor case with quantified load-sensitive cognitive–language–gait dysfunction and to detail a physiology-guided, sequence-driven decompression approach emphasizing ventricular relaxation and perforator-preserving, interface-limited thalamic resection. **Case Presentation:** A 56-year-old female patient experienced a 3-month, rapidly progressive decline in her cognitive and language abilities. The clinical progression was not stepwise or punctuated by a single “sentinel” event. She had a moderate level of cognitive impairment consistent with both Broca’s and Wernicke’s aphasias (MoCA: 22/30) and suffered from significant interference effects and increased cost of task-switching. Her ability to generate novel responses and name objects was significantly impaired; however, she was able to repeat words and phrases appropriately. In addition, she exhibited a severe sustained attention signature and a high error rate during dual-task performance, indicating severe gait instability, although her overall global anchors were nearly neutral (GCS 15; FOUR 15/16; NIHSS 2). Nausea and vomiting occurred simultaneously with the cognitive and language decline, suggesting decreased intracranial compliance. MRI revealed a heterogeneous left-sided thalamic tumor extending into the posterior horn of the lateral ventricle. The tumor caused deformation of the lateral ventricle and midline displacement. The patient underwent microsurgical intervention using a physiology-conscious sequence of graded cerebrospinal fluid (CSF) equilibration and primary mechanical removal of the tumor from the ventricular system. Additionally, decompression of the thalamus was performed in a manner that was cognizant of the boundaries formed by the perforating arteries of the thalamus. Early resolution of pressure symptoms was noted postoperatively. Objective measures demonstrated significant improvement in the patient’s executive functioning, language skills, attentional errors, and dual-task performance stability. The patient remained functionally independent at discharge and at subsequent follow-up visits. Surveillance imaging did not demonstrate any evidence of tumor recurrence. **Conclusions**: The clinical presentation described above is supportive of a model in which the synergy between deep network damage and distortion of the posterior ventricular compartment amplifies network dysfunction. Additionally, the use of quantitative stress-phenotyping makes it possible to identify deep network pathology early in its course. Finally, the physiology-guided decompression approach that was used in this case has the potential to increase functional reserve in patients with pathology that requires millimeter transitions.

## 1. Introduction

Physically, where lesions occur within periventricular (PV) regions of the brain and deep thalamus, relative to the cerebrospinal fluid (CSF) space and the shapes/structures of the ventricles, may result in differing effects on intracranial compliance and CSF buffering compared to areas distant from the ventricular system. Therefore, the physical positioning of PV and deep thalamic lesions relative to the CSF space and the ventricular geometry can produce clinically relevant effects on the brain’s ability to accommodate pressures and its capacity to buffer CSF in the respective CSF spaces, regardless of the size of the lesions [1,2].

Deep thalamic tumors constitute less than 5% of all adult intracranial tumors; however, a single deep thalamic tumor can cause global alterations in the regulation of thalamic-gated activity in the cortex and in executive functioning and attention. Often, the global disruptions caused by such tumors are obscured by the presence of a focal clinical presentation [3]. As the PV portion of a tumor compresses the ventricular atria and posterior horns, thereby altering the local flow of CSF through the space, a pressure-perfusion mismatch can develop, resulting in a rapid deterioration of the patient’s clinical status, despite the small volumetric burden of the lesion [4].

Theoretically, thalamocortical systems have evolved from a simple relay model to a more complex model of network dynamics. Therefore, injury to specific thalamic nuclei and/or the PV association fibers surrounding the nuclei (which include those related to executive function, language, and posture) can result in slowed processing speeds, decreased ability to inhibit irrelevant stimuli, decreased ability to shift between tasks, and an inability to perform two tasks simultaneously before a decrease in motor strength [5]. Cognitive inefficiency, retrieval-weighted language loss, and gait instability when loaded can serve as indices of compromised deep networks in individuals who test normal on standard screening tests. Additionally, alterations in the compliance of the ventricles and the resultant changes in the shape of the ventricles can worsen the inefficiencies of the deep networks, while stimulating pressure-dependent autonomic responses that can give rise to a “pressure-network” phenotype that is distinct from general cognitive impairment or isolated intracranial hypertension [6]. Clinical experiences and case reports have demonstrated that patients who have deep lesions extending into the ventricle and later develop symptoms such as nausea, vomiting, fatigue-related cognitive collapse, and diurnal variability have impaired CSF buffering and compartmental stress rather than widespread cortical edema [7]. Such findings indicate that the clinical phenotype, the shape and geometry of the ventricle, and the physiological reserve of the individual are interrelated variables [8]. Operative strategies to remove tumors located in the deep thalamus and intraventricularly are complicated by the high perfusion rates of these structures and the dense packing of nerve tracts. Due to the geometry of the structures, minimal geometric changes can lead to significant functional consequences. Rapid decompression of these tumors can also disrupt the subependymal venous system and create an unfavorable pressure-perfusion gradient [9]. Therefore, operative techniques to remove deep thalamic tumors are developing to treat the ventricular system as a dynamic physiological compartment by providing for slow CSF equilibration, defining boundaries during tumor removal, and preserving the thalamic interface to allow for safe decompression of the tumor without jeopardizing the thalamic perforators [10].

Although there is an ever-increasing amount of literature being published about the network phenotypes, compartment anatomy, operative techniques that incorporate physiological aspects, and long-term follow-up, most of the current literature is relatively small-scale. Even well-characterized case reports of a single patient can provide valuable mechanistic insight if a clear clinic-anatomy-physiology relationship can be established [11]. The purpose of this report is to present a patient with a deep thalamic lesion that extends into the posterior compartment of the ventricle and illustrate how network-dominant cognitive/language dysfunction and dual-task gait instability developed due to thalamocortical disruption that was exacerbated by the low compliance of the ventricular compartment. We also detail a physics-based operative technique and postoperative care that used gradual pressure equilibration and preserved the thalamic boundary rich in perforators and discuss a methodological approach to monitor the development of the phenotype over time.

In summary, we propose that in the case of deep thalamic-intraventricular lesions, the degree to which the clinical condition deteriorates will depend on the physiological reserve of the affected network(s) and the compliance of the craniospinal compartment, in addition to the volume of the lesion. Documenting these parameters in a standardized fashion provides a transferable template for evaluating and treating patients with similar clinical syndromes.

Clinical Relevance and Knowledge Gap

A major clinical limitation of previous studies in this area is the lack of a universally accepted, easy-to-understand, and reproducible way to correlate anatomical and physiological concepts to the operative strategy. Some examples of unanswered questions include: How do the geometric configurations of the pressure-coupled thalamo-ventricular relationship influence the timing and sequence of the decompression? What intraoperative physiological milestones should direct the staged decompression? Where along the interface should the resection cease, and why, both technically and physiologically? This issue is important clinically because many patients will have undergone routine screening and motor-weighted testing that were unremarkable until the load-sensitive networks began to fail, and the window for early intervention closed to preserve physiological reserve.

## 2. Case Presentation

The 56-year-old woman has a history of memory loss that is progressive and worsening over the last three months. Additionally, she has shown a decline in her ability to express herself verbally for the same three-month period with no apparent cause, such as a stroke or traumatic head injury. The patient’s score on the Montreal Cognitive Assessment (MoCA) of 22/30 suggests that the cognitive dysfunctions associated with her condition are more likely to be due to dysfunction of the load-sensitive network as compared to localized damage to one area of the brain. Although the patient could complete moderately complex tasks independently, the large amount of stimuli required to complete these tasks, along with the high degree of cognitive processing required to complete these tasks, severely impacted the patient’s ability to communicate effectively and significantly hindered the patient’s ability to complete these tasks. As such, the general pattern of dysfunction observed in the patient was thought to be consistent with a throughput-limited syndrome of executive function, lexical retrieval, and balance regulation due to the disruption of the strategic integrative hub or long-range connectivity patterns, as opposed to inflammatory encephalopathy or primary neurodegeneration.

T0 Phenotyping: Evaluating the Impairment of Network Function During Stressful Cognitive Processing (Pre-Imaging)

A more detailed characterization of the impression obtained through the initial screening process was made using a targeted load-stress battery administered to the patient at T0 to measure the “failure signature” observed in all tested areas. This battery evaluated executive function and the efficiency of cognitive processing, both of which were impaired (SDMT = 28; FAB = 12/18; ACE-III = 78/100). An abnormal degree of susceptibility to interference was noted (Stroop Color-Word = 48 s with 8 errors; Stroop interference cost = +22 s). Similarly, the cost of switching sets was substantially inflated (TMT-A = 35 s, 0 errors; TMT-B = 128 s, 4 errors), indicating that the mechanisms used to stabilize top-down processes had become exhausted and that the impairment was not simply a result of non-specific slowness. Language testing of the patient revealed a retrieval-weighted deficit, while the repetition portion of the test was spared, indicating that retrieval/mapping impairments under conditions of increased demand versus deficits in motor speech were present (BNT = 42/60; Phonemic Fluency = 9/60 s; Semantic Fluency = 14/60 s; Repetition = 10/10; Comprehension = 8/10).

In addition, the patient experienced nausea and vomiting during the period of decline in function, indicating that exhausted intracranial compliance and pressure-mediated symptomatology were present. The preservation of the global neurological anchors (GCS = 15; FOUR = 15/16; NIHSS = 2; Dysarthria) permitted a clinically meaningful separation of preserved arousal/strength from failing higher-order control processes. Sustained attention demonstrated an error-dominant profile (d2 total = 287; d2 concentration = 112; d2 errors = 18), and therefore reduced output fidelity under sustained throughput. Executive-postural decoupling was demonstrated via mobility assessments as follows: the patient’s baseline timed-up-and-go (TUG) times were less than average (12 s); however, the imposition of cognitive loading resulted in a significant destabilization of postural control (Dual Task TUG = 28 s with serial subtraction errors = 5), and thus postural control was compromised at an integrative limit when executive resources were diverted.

Phenotype-Guided Differential Prioritization (Prior to Imaging)

Due to the relatively short duration of the patient’s illness and due to the patient’s nausea/vomiting secondary to pressure effects, the likelihood of common neurodegenerative syndromes was reduced. Due to the patient’s cross-domain control failures and due to the patient’s aphasic phenotype being structurally consonant and the repetition component of the patient’s language testing being preserved, functional/psychiatric etiologies were de-prioritized. Due to the clinical progression of the patient’s illness being characterized by pressure effects as opposed to fluctuating diffuse cortical irritation, inflammatory/metabolic encephalopathies were considered to be less parsimonious. Laboratory tests were also performed to confirm procedural readiness and intact coagulation (INR = 1.1; aPTT = 28 s; Fibrinogen = 345 mg/dL; D-Dimer = 320 ng/mL; TEG R = 5.2 min; MA = 62 mm), and thus common causes of metabolic encephalopathy were excluded. The patient did not demonstrate a stepwise decline typical of ischemic stroke. Disorders involving shunt physiology, predominantly arterial/venous obstruction, and the hydrocephalus spectrum were also considered but did not adequately explain the combination of interference susceptibility, switching cost inflation, and retrieval-weighted aphasia.

Thus, a deep space-occupying lesion was considered to be the most probable diagnosis prior to imaging to explain the following four characteristics: (i) Significant executive control failure under conditions of high throughput; (ii) a retrieval-weighted aphasia with preserved repetition; (iii) extreme dual-task gait destabilization indicating control limits; and (iv) nausea/vomiting indicating exhausted intracranial compliance. Potential lesion types include: (i) Infiltrating glioma, (ii) primary CNS lymphoma, (iii) metastasis, and (iv) tumors located near the ventricles, each of which implies different diagnostic and decompression trade-offs between oncologic control and network preservation.

Cerebral CT scans revealed a mass located in the left thalamus extending into the left posterior horn of the lateral ventricle with a bi-component lesion (nuclear and intraventricular). On non-contrast CT, the mass was seen as hypodense with heterogeneous areas of enhancement seen on post-contrast CT scans, indicating internal heterogeneity within the mass. Ipsilateral ventricular distortion and a 1 cm midline shift to the right were present, indicating exhaustion of intracranial compliance and anatomical distortion of the ventricular system contributing to the patient’s symptoms of nausea and vomiting.

**Figure 1:**
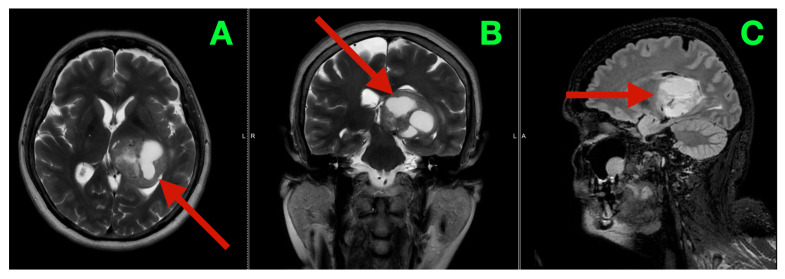
Preoperative MRI (non-contrast; parenchymal substrate and compartmental geometry). (**A**) Axial T2-weighted image demonstrates a heterogeneous left thalamic lesion (red arrow) with mixed internal signal intensity and direct extension into the posterior horn/atrial region of the left lateral ventricle, producing distortion of adjacent periventricular anatomy without diffuse cortical edema. (**B**) Coronal reconstruction highlights the deep midline-adjacent position of the lesion (red arrow), emphasizing its relationship to the thalamic body and the ventricular atrium, a region densely traversed by perithalamic and long-association fiber systems relevant to executive and language integration. (**C**) Sagittal view delineates the cranio-caudal extent of the lesion (red arrow) and its intraventricular projection, illustrating the dual-compartment geometry through which a deep nuclear process can exert both network-level disruption and pressure amplification via CSF pathway distortion.

**Figure 2:**
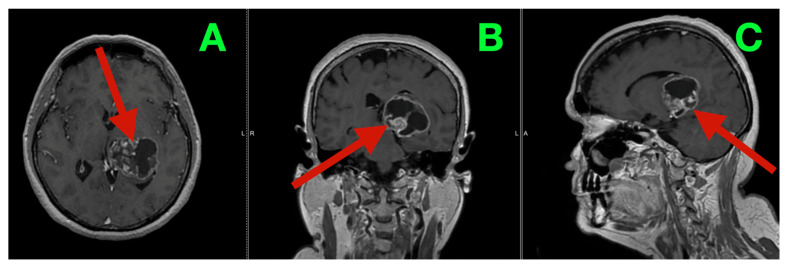
Preoperative MRI (post-gadolinium T1-weighted; biological architecture and interface definition). (**A**) Axial post-contrast image demonstrates irregular, heterogeneous enhancement of the left thalamic lesion (red arrow), distributed along complex internal interfaces rather than forming a uniform enhancing mass, consistent with spatially variable blood–brain barrier disruption. (**B**) Coronal post-contrast image emphasizes the lesion’s enhancement pattern at the thalamic–ventricular junction (red arrow), supporting a biologically aggressive process with mixed microenvironmental compartments rather than a homogeneous low-grade lesion. (**C**) Sagittal post-contrast view further defines the lesion’s deep epicenter and intraventricular extension (red arrow), providing anatomical context for the network-dominant clinical phenotype and the pressure-sensitive evolution of symptoms.

MRI provided additional detail regarding the morphology of the lesion and its substrate. T2-weighted images (Figure 1A–C) showed a mixture of internal hyperintensity and hypointensity, with multiple solid/cystic/necrotic interfaces within the mass. These findings supported that the mass contained no single cyst and that it was composed of a variety of low-grade lesions. The center of the mass was at the thalamo-atrial/posterior horn interface and accounted for the patient’s clinical presentation. The thalamus serves as a node-gate for state transitions in networks, and the atrial periventricular region contains a large number of association/perithalamic pathways involved in processes such as executive set formation, attentional economy, language control, and posture. Post-contrast T1-weighted images (Figure 2A–C) showed variably sized and shaped patches of enhancement along the inner margins of the mass, indicating spatially varying disruption of the blood–brain barrier.

**Figure 3:**
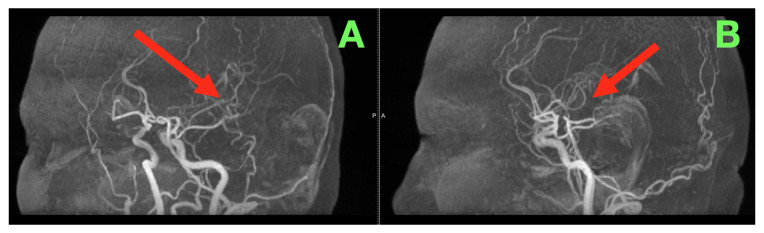
Preoperative vascular-context imaging (arterial geometry and hemodynamic exclusion). (**A**,**B**) Lateral vascular projections demonstrate preserved intracranial arterial continuity and geometry in the region adjacent to the lesion (red arrow), without evidence of early venous filling, arteriovenous shunting, or dominant vascular flow patterns that would suggest a primary hemodynamic driver. The vascular context supports a mass-effect–dominant and infiltrative framework, rather than a shunt-mediated mechanism, for the patient’s clinical presentation.

Considering the size of the mass (4–5 cm), the extent of the involvement of the ventricular system, and the pattern of enhancement of the mass, an active gliomatous process was felt to be the most plausible diagnosis. Considering the mass’s relationship to the major vascular structures and the physiologic context of this case, vascular context imaging was performed to determine whether a shunt-related physiology or significant arterial/venous restriction existed that might alter either the mechanism of injury or operative risk. The vascular studies (Figure 3A,B) demonstrated preserved anatomy of the major arteries and veins, absence of early venous filling, and absence of high-flow arteriovenous transit, indicating that the mass effects/infiltrative mechanisms of the mass were primarily responsible and not due to a primary shunt-mediated hemodynamic mechanism.

Anatomy-to-Operative Goals Based Upon Physiology:

The anatomy and the position of this lesion created a strategic hub-disruptor and a posterior ventricular “pressure amplifier.” The combined anatomy of the deep thalamic and intraventricular components likely contributed to the rapid and measurable network collapse and the pressure-dependent nature of the patient’s symptoms. As such, the oncology-oriented and physiologically oriented goals of the surgery were: (i) to disrupt mass–CSF coupling at the atrial-posterior horn junction; (ii) to obtain representative tissue; and (iii) to decompress the thalamic boundary while preserving the perforator-rich, fiber-dense periventricular corridor. The limitations of the procedure were: (i) Access; (ii) Maintaining physiological homeostasis during geometric transitions; (iii) gradually decompressing; (iv) controlling hemostasis; and (v) treating the tumor–thalamus interface as a functional boundary and not a margin-based target.

Workflow of the Operative Procedure: Transcortical-Transventricular Decompression in Sequence and Guided by Physiology:

In order to facilitate reproduction of the procedure, we converted the procedure into a step-by-step workflow with restoration of ventricular compliance and integrity of the tumor interface serving as explicit intraoperative checkpoints.

Creation of Corridor and Positioning:

The patient was placed so that a straight line existed from the cortex to the ventricles and to avoid potential venous outflow obstruction and perforator displacement around the ventricle. A custom craniotomy was designed to create a transcortical-transventricular corridor to allow visualization of the ventricular system as the primary physiological space. Following the opening of the dura, a microscopic cortical entry was made into a zone that was relatively safe from risks and was protected circumferentially with intact pia mater, where precise hemostasis was accomplished with minimal cortical coagulation. The corridor was dynamically maintained through internal decompression to minimize the amount of traction on the ventricular wall.

Technical establishment of the transcortical–transventricular corridor:

Use of preoperative MRI and intraoperative neuronavigation enabled us to design the most direct approach possible to the posterior horn of the lateral ventricle/atrium from a cortical entry site; we thus avoided the deeper brain structures that were most likely to be damaged had we continued to expose the cortex (e.g., motor cortex, sensory cortex, and association cortex) and reduced our risk to the cortical language areas. To optimize the positioning of the patient so that the intended trajectory could take place in a gravitational plane (thus making exposure easier and reducing the possibility of compromising venous drainage), the patient was positioned on the frame such that their head was in the appropriate position. Using the data derived from the preoperative navigation, a customized frontal craniotomy was performed above the anticipated entry site, and the dura was opened to allow a microsurgical corridor to be developed with the least amount of cortical damage. Utilizing high-power magnification, a cortical entry site was created utilizing a corticotomy that was less than 1.5 cm in length and oriented parallel to the orientation of the cortical fibers. Bipolar coagulation was employed to achieve hemostasis at the cortical entry site at the level of the individual surface/pial vessels, and limited thermal injury was accomplished to the surrounding cortex. Following the development of a subcortical pathway employing bipolar tips and suction as a dissector to separate the white matter along white matter planes, the surgeon employed both handheld dynamic retraction and gravity to maintain the integrity of the operative corridor throughout the passage to the subcortical region. Neuronavigation was again utilized prior to entering the ventricle, and entry into the ventricle was made with deliberate and precise movements to minimize the risk of blind suction. In this case, no tubular retractor was employed to ensure the patency of the corridor; the ventricle served as the primary operative working space, and it was maintained by means of continuous internal decompression and periodic relaxation pauses.

Checkpoint 1: Gradual Equilibrium of CSF:

Following entrance into the ventricle, CSF was gradually released over approximately 90 s to prevent sudden shifts in compartmental pressures. Once the progressive reduction in tension of the ventricular wall and subsequent relaxation of the ventricular wall towards a nearly normal contour had been achieved, the physiological threshold for initiation of intraventricular debulking had been attained.

Step 1: Ventricular First Inside-Out Decompression:

Since the intraventricular component of the mass was believed to make a significant contribution to the compartmental pressure, it was the initial target of the debulking efforts. Using high magnification, the tumor was found to consist of two distinct portions: (i) an exophytic intraventricular component (soft, fragile, and very vascular) that could be removed with ease using low-power suction/ultrasonic aspiration and (ii) a thicker, less vascular thalamic base with a greater resistance at the junction. Central debulking began and progressed outward to allow for gradual collapse before external manipulation and to minimize the amount of traction exerted on the ventricular wall. Due to adhesions to the thickened, highly vascular ependyma of the pulvinar, the medial and superior aspects were removed with caution. Since the ventricular wall contained many periventricular association tracts in the atrium/posterior horn, reducing traction was a procedural constraint.

Structured inside-out decompression sequence:

The “ventricle” has been described as the “work area.” All of the decompression process will take place “internally,” or inwardly, utilizing the least amount of negative pressure via a combination of micro-suction and an ultrasonic aspirator to first remove the softest portion of the tumor located in the center. Once sufficient space exists within the ventricle for the tumor to move toward the interior, the formerly exophytic mass with traction potential becomes less adherent and is now supported internally by the force of the tumor’s collapse toward the interior. Once the interior portion of the tumor collapses, the remaining margins of the tumor are removed using alternating quadrant debulking procedures to prevent unbalanced traction forces on the ependymal wall. The ependyma may be identified based on a shiny appearance and a clearly visible border between the ependyma and the tumor (color and texture). Rather than employing avulsion to free the adherent segments of the ependyma, these are removed using an irrigation technique. With the cessation of each microdebulking procedure, the flow of irrigation is stopped temporarily, and the physiological changes that occur in the wall of the ventricle and cerebrospinal fluid pulsations are monitored to assess whether or not compliance of the ventricle has been regained prior to the next procedure.

Venous Preservation and Hemostasis:

Continuous irrigation and fine bipolar coagulation were used to achieve hemostasis, limiting thermal spread; extensive surface coagulation was avoided. The perforating venous structures, including the thalamostriate vein located on the floor of the ventricle, were preserved.

Thalamic–tumor interface identification and protection:

The thalamus was viewed by neurosurgeons as an interface rather than a margin requiring dissection; therefore, the surgeon assessed for the presence of multiple interface cues as described above and observed these cues continuously through a microscope to assess the presence of all of the interface cues. At low magnification, the surgeon could observe an increase in aspiration resistance, disappearance of intra-tumoral heterogeneity, and appearance of organized tissue; at higher magnifications, a change in color toward a homogenously vascularized parenchymal pattern and an increasing number of fine perforating vessels, which were parallel to the dissection plane. When those interface cues were present, the surgeon switched from a suction-based method of removing the tumor to using microdissecting techniques to remove the tumor while simultaneously performing internal debulking. The ependymal layer served as the constant reference point for the surgeon during this process. For controlling pinpoint bleeding caused by bipolar coagulation, the surgeon used continuous irrigation to minimize the thermal spread associated with bipolar coagulation. Traction on adherent structures was usually avoided, particularly in the approach to what has been called the “anterior inferior corridor,” because the surgeon recognized the potential for the posterior limb of the internal capsule and/or optic radiations to have been involved in the tumor. In addition to traction avoidance, deep dissections were performed in micro-cycles (internal debulk → re-orient on ependyma → assess presence of all of the interface cues → continue only if it is safe) so as to allow the surgeon to continually assess whether or not it was safe to proceed with further dissection. A ≥2 cue stop rule was developed by the surgeon to establish the formal endpoint of resection when the thalamic proximity/perforator risk became predominant.

Step 2: Interface-Limited Thalamic Decompression:

After achieving ventricular relaxation and tumor collapse, the focus of the procedure shifted to the thalamic interface. The ultimate goal of the operation was to anatomically and functionally define the points of termination of dissection in the thalamus.

Checkpoint 2: Defined Stop Criteria (Interface Endpoints):

Dissection of the thalamus was stopped when evidence of proximity to functional thalamic parenchyma and increased perforator risk were detected: (1) Increased suction/aspiration resistance; (2) transition from soft, amorphous tumor to solid, organized brain tissue; (3) detection of small intrinsic vessels within approximately 1 cm of the deep margin, indicative of perforator density; and (4) loss of internal heterogeneity allowing for clear distinction between tumor and parenchyma. If ≥2 criteria were met, further resection of the deep part of the tumor was ceased, and maximum ventricular decompression was completed without further damage to the thalamus. The final deep margin was the posterolateral thalamus; the anteroinferior border was approached but not entered due to proximity to the posterior limb of the internal capsule and surrounding optic radiations. All small perforators encountered were preserved, and continuous high-precision coagulation was carried out under irrigation. Debulking occurred in micro-steps (debulk → re-orient along ependyma → assess deep margin → repeat), therefore minimizing entry into the functional thalamus while maximizing decompression.

Exposure, Verification, and Closure Strategy:

Bone retractors were avoided, and gravity and dynamic microsurgical retraction with minimal sustained pressure were employed. After debulking, the cavity was inspected visually under normotensive conditions to locate any residual fragments that may cause subsequent ventricular deformity. The ventricular walls had significantly relaxed toward a normal contour, and CSF pathways were visible with mild pulsatile flow. The closure was watertight; the bone flap was securely fixed to avoid secondary compression, and a layered closure was accomplished.

**Figure 4:**
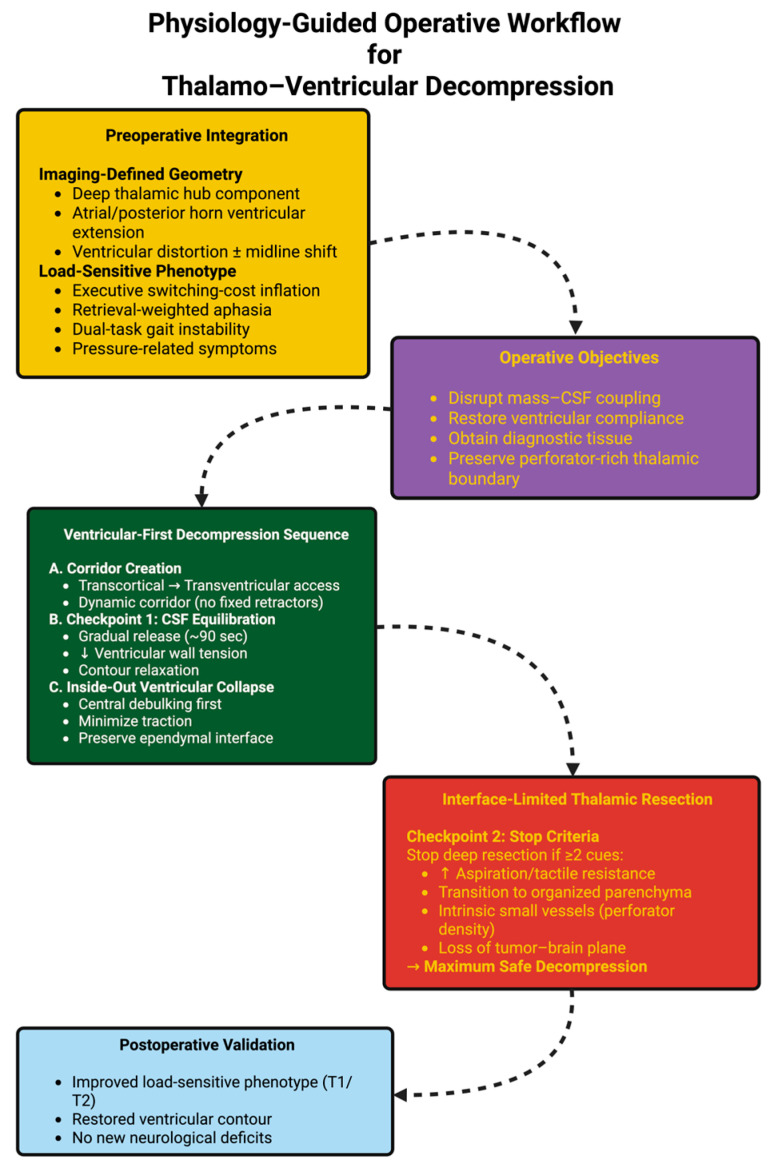
Algorithm linking preoperative geometry and load-sensitive phenotype to a sequence-driven strategy: graded CSF equilibration → ventricular-first inside-out decompression → interface-limited thalamic resection with ≥2-cue stop rule. The endpoint is maximum safe decompression with postoperative validation.

Summary of Operative Procedure:

The rationale for the operative procedure was the following: (i) Gradually equilibrate CSF → Ventricular-first internal decompression → Interface-limited thalamic decompression with preservation of perforators and subependymal veins to restore intracranial compliance and relieve deep hub stress while creating minimal new neurological deficits. To improve clarity and reproducibility, we summarize this physiology-guided, sequence-driven operative logic as a decision-based workflow with explicit checkpoints and stop criteria (Figure 4).

The main objective of postoperative management of this patient was to protect the very fragile thalamo-intraventricular tract from being damaged when the pressure changed from a high to a low-pressure state and to prevent instability of the ventricular compliance due to the acute damage to the subependymal veins and delay in the development of perilesional edema. Upon awakening, the patient was awake, cooperative, and hemodynamically stable with normal levels of consciousness (GCS = 15/15; FOUR = 16/16). The evaluation of the potential for a deep network failure, which may have been missed by a motor-weighted screening method, and the degree of global cognitive decline, as well as the degree of autonomic function affected by pressure (NIHSS = 1; mild dysarthria), was part of the post-waking evaluation.

Three-anchored frame (n = 1 repeated measures): Three assessments were performed at T0 (pre-surgical baseline measurement), T1 (short-term post-operative; POD 0–POD 3), and T2 (follow-up 8 weeks after surgery). All data were reported as raw data and as change data in order to report similar physiological responses across patients, as opposed to reporting statistical trends.

T1: Short-term evaluation in the post-operative time period. Early language evaluations performed in the post-operative period demonstrated preserved comprehension, repetition, and spontaneous speech, and enhanced lexical access (Comprehension = 10/10; Repetition = 10/10; BNT = 48/60; phonemic fluency = 13/60 s; semantic fluency = 18/60 s). Additionally, improvements in executive functioning and processing speed were observed (MoCA = 26/30; SDMT = 35; FAB = 14/18; ACE-III = 86/100). Improved load sensitivity was also observed, as evidenced by improved interference control (Stroop = 38 s; 4 errors; interference +12 s) and set shifting (TMT-A = 32 s; 0 errors; TMT-B = 98 s; 2 errors). Improved sustained attention and output fidelity were also observed (d2 total = 312; concentration = 128; errors = 11).

**Figure 5:**
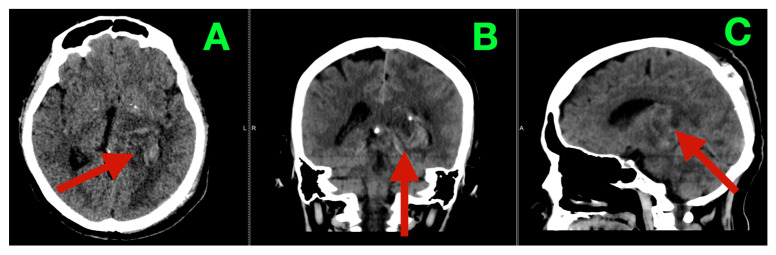
Postoperative day 7 non-contrast CT (surveillance of operative bed and compartmental evolution). (**A**) Axial reconstruction demonstrates the left thalamic–atrial/posterior horn operative cavity (red arrow) with expected postoperative configuration, assessed for hemorrhage, evolving edema, and ventricular patency. (**B**) Coronal reconstruction delineates the deep periventricular topography of the operative bed (red arrow) and its relationship to the lateral ventricle, supporting evaluation for obstructive hydrocephalus or progressive compartmental distortion. (**C**) Sagittal reconstruction illustrates the cranio-caudal extent of postoperative change along the thalamic–ventricular interface (red arrow), serving as an objective anchor for interval mass-effect and compliance trajectory.

Physiological management. Hemodynamic monitoring was used to maintain stable blood pressures (SBP = 120–140 mmHg; MAP = 80–90 mmHg). Dexamethasone 4 mg q6h was tapered over a 5-day period to reduce cerebral edema. Levetiracetam 500 mg BID was administered for seizure prophylaxis. Minimal nausea (NRS = 2/10 POD 0; 0 vomiting episodes) was experienced by the patient. Stable laboratory values were maintained throughout the post-operative period, providing evidence of clinical stability (Hb = 11.8 g/dL; Na = 138 mmol/L; Glucose = 112 mg/dL; INR = 1.0; aPTT = 27 s; Platelets = 245 × 10^9^/L; CRP = 1.2 mg/dL; D-dimer = 450 ng/mL). Although prospective volumetric and compliance measurements were not collected, post-operative imaging provided an anatomical basis to assess compartmental status and corridor-related complications. A non-contrast CT scan was taken on POD 7 (Figure 5A–C), demonstrating a small localized cavity at the thalamus—atrial/posterior horn interface with preserved ventricular contour and no hemorrhage, mass effect, or obstructive hydrocephalus.

Clinical Course and T2. Continued improvement in cognitive and linguistic efficiency occurred until POD 3 (MoCA = 27/30; SDMT = 38; FAB = 15/18; ACE-III = 89/100; Stroop = 35 s; TMT-A = 30 s; TMT-B = 85 s; d2 errors = 8; NRS = 0/10). By POD 1, the patient had stopped experiencing nausea and vomiting. Improved executive-postural coupling and less dual task penalty were observed (TUG = 10 s; Dual Task TUG = 15 s; Serial Subtraction Errors = 2). The patient was discharged independently (mRS = 1; Barthel = 95/100) without new focal motor deficits and with conversational ability. The patient’s discharge medications consisted of levetiracetam 500 mg BID and a completed dexamethasone taper.

At eight weeks (T2), the patient had full resolution of her pressure-related symptoms, and she was capable of performing higher-level cognitive tasks. Global cognition and frontal regulation were almost back to normal (MoCA = 29/30; FAB = 17/18; SDMT = 41). Increased resistance to interference (Stroop = 32 s; interference +8 s), increased set-shifting (TMT-B = 72 s), and increased lexical retrieval (BNT = 58/60; Phonemic Fluency = 17; Semantic Fluency = 22) were observed. Fewer attention errors (d2 errors = 5) and equal dual-task mobility (TUG = 9 s; Dual Task TUG = 11 s) were observed. Full symptom resolution, measurable increase in load-sensitive integration, and post-operative imaging evidence of stable compartments support the conclusion that decompressive surgery utilizing a physiology-guided, interface-limited approach was successful in restoring functional reserve in this configuration.

Synthesis

It is clear, based on the preoperative characteristics of the patient and dual-compartment anatomy, that the lesion served as both a strategic hub disruptor and a posterior ventricular pressure amplifier. Therefore, the surgical strategy addressed both tissue diagnosis/cytoreduction and the restoration of compliance by disrupting mass-CSF coupling at the atrial/posterior horn junction, while preserving the integrity of the thalamic border, periventricular associations, and perforator-rich areas. The quantification of the patient’s load-sensitive phenotype, followed by a target defined by geometric analysis and checkpoint-driven decompression, represents the primary technical innovations of this case.

## 3. Discussion

The key difference between deep thalamic tumors that can either enter the atrium or posterior horn of the lateral ventricle, thereby disrupting the compliance of the ventricle and the fluid dynamics of CSF, and “simple” deficit-producing tumors lies in the ability of deep thalamic tumors to act as both deficit producers and as integrators. Because small geometric distortions at the juncture of the thalamus and the ventricle can produce deficits in both the gating of thalamic hubs and the compliance and CSF buffering capability of the ventricle, symptoms are likely produced by a combined mechanism of disrupted thalamocortical gating, disruption of perithalamic long association coupling, and pressure/stress generated by distorting CSF pathways and by failing compliance in the immediate area around the ventricle [3]. Clinically observed to merge into a syndrome of inefficient execution characterized by higher than normal costs for switching from one task to another, impaired retrieval-weighted language skills, destabilized gait with increasing load during performance of dual tasks, and nausea/vomiting representing exhausted compliance rather than focal damage alone [12]. Our case provides evidence supporting this mechanism by demonstrating a measurable, load-sensitive phenotype resulting from a two-compartment geometry of the thalamus and the atrium/posterior horn of the lateral ventricle and by documenting significant recovery after decompression.

A radiographically demonstrable thalamic tumor that involves the ventricle with heterogeneously enhanced imaging characteristics is radiologically indistinguishable from a number of other entities, including glioblastoma, adult diffuse midline glioma (DMG), and inflammatory/lymphoid mimics such as primary central nervous system lymphoma [13] and the diagnosis relies heavily upon the biological features of the tumor [14]. Biological adjudication is particularly important because therapy/prognosis for these tumors increasingly depends upon the molecular classification of the tumor rather than its imaging characteristics [14].

Operationally, the primary technical challenges of surgical approaches to these tumors relate to the proximity of the perforator/fiber-dense interface that encircles the subependymal veins. While the ventricle can provide a physiological workspace, it significantly increases the vulnerability of the brain to traction, venous destabilization, and acute pressure changes. Therefore, the operative strategy is often sequence dependent (i.e., based on progressive steps that reflect the natural history of the disease process) rather than margin dependent (i.e., based on the extent of removal of all grossly visible tumor) [15]. The novel aspect of this approach does not relate to the degree of aggressiveness employed but to converting “physiology guided” intent into a practical operating procedure with checkpoints and stopping criteria: graded CSF equilibration as an early compliance checkpoint; ventricular-first inside-out collapse to restore relaxation and minimize traction; and interface limited thalamic decompression based on multiple, converging stop cues (e.g., rising aspiration/tactile resistance, transitions in parenchymal texture indicative of increasing proximity to small vessels consistent with perforator density, and loss of discriminable planes; ≥2 cues indicate cessation) [16]. Thus, “maximum safe resection” becomes maximum safe decompression within a functional, anatomical boundary.

This approach is most applicable to patients who meet three conditions: (1) the tumor has deformed the atrial/posterior horn compartment of the ventricle in a functionally meaningful manner; (2) the patient’s phenotype is load sensitive to a greater degree than would be predicted by their motor weighted anchors; and (3) pursuit of margin would predictably result in increased risk at the perforator-rich interface [17]. Patients whose tumors do not involve ventricular extension/compliance stress or whose biology warrants a more aggressive oncologic approach will require the use of a different set of stopping rules [18].

The framework recognizes the extremely high cost of millimeter-scale errors in areas with low redundancy, not a call for radicality [19]. The context of this case study within the current body of literature regarding the diagnosis and treatment of deep thalamic-intraventricular tumors was provided through the synthesis of representative original and translational studies (Table 1) related to the biology and clinical management of these tumors.

Diagnostic and postoperative management of patients with deep/midline gliomas will similarly require integration of the biology of the tumor with recovery of networks affected by the tumor. Assessment of CSF/plasma liquid biopsies for deep/midline gliomas is currently being explored, while developing proteogenomics of H3K27-altered DMG shows pathway-level and immune signature complexities beyond single mutations [31]. Therefore, integrated classification (histology + methylation class + targeted sequencing ± CSF assays) is becoming increasingly important for midline tumors and postoperative management should strive to strike a balance between oncologic control (maximal safe cytoreduction ± radiotherapy/chemotherapy based on markers such as MGMT) and mechanistic support (steroid taper, antiseizure prophylaxis, and tight hemodynamic control) to protect vulnerable periventricular tissue and facilitate recovery of networks [32,33].

Reporting of responses to targeted therapies in selected H3K27-altered DMG cases also suggests that treatment options will become increasingly dependent upon accurate diagnostic characterization [34].

While this report represents a single-patient mechanistic demonstration and should not be interpreted as implying population-wide effects, vascular context imaging cannot be conclusively correlated with physiologic measurements in this report. Volumetric analysis, quantitative ventricular deformation, and direct compliance measurements were not performed prospectively; thus, post-operative imaging served as the structural reference for the bed configuration and ventricular patency. Although formal analysis is lacking, this case clearly illustrates coherent convergence across levels—load-sensitive executive/language dysfunction with dual-task instability, pressure-coupled thalamo-ventricular geometry, explicit intraoperative stop rule logic, and persistent improvement in function post-decompression. A prospective series incorporating standardized stress phenotyping, explicit intraoperative endpoints, and quantitative compartment metrics will be necessary to assess generalizability and to identify which factors best predict stable recovery.

## 4. Conclusions

When large thalamic tumors push against the back of the brain’s ventricles, the limited space they occupy can compress many symptoms into a few: even small changes in the depth of structures within the brain can cause large changes in the speed of thinking, the precision of language use when under load, and the ability to maintain stable gait while performing two tasks at once. Thus, the narrative of a bedside examination (i.e., physical examination of the patient) may not be complete without additional data to describe the specific ways in which function fails, in addition to identifying deficits using a broad screening test. This patient’s post-operative course was suggestive of the idea that surgeons should consider the ventricle as a physiologic unit that acts as part of the brain (and not just a reference to the location of the ventricle) and develop treatment plans accordingly. We sought to combine a microsurgical decompression plan based on the anatomy and physiology of the area (specifically the ventricles) with a monitoring strategy that would utilize objective network metrics as surrogate markers to track the progression of recovery and provide evidence that recovery occurs as an increase in the amount of functional margin available in the brain (as opposed to the presence or absence of new focal deficits). The correlation we found between the stability of the anatomical compartments involved in the surgery (as evidenced by imaging), and the improvement seen in both executive-language and dual-task mobility metrics provides a practical method for determining the benefits from deep midline surgery when the primary goal is not radical surgical removal but instead the preservation of all viable tissues; in particular, the distinction between the amount of tissue removed and the classification of tissue removed, are likely to determine the timing and nature of the next therapeutic option.

Our aim in presenting this case study is to contribute a simple yet applicable paradigm to guide the planning and execution of future cases: mechanism-focused phenotyping to improve the specificity of inferences made prior to surgery, physiology-based microsurgical approaches to minimize injury from the surgeon’s instruments to vulnerable areas of the brain, and monitoring post-operatively to evaluate the rate of recovery of connectivity in the brain (using quantitative metrics), as opposed to relying solely on subjective reports of improvement or worsening of symptoms. As the field continues to evolve with the development of methylation-based classifications of tumors, CSF-based molecular profiling of tumor cells, and targeted therapies for various types of midline lesions, an approach such as the one described above will allow for improved coordination of diagnosis, treatment, and assessment of outcomes throughout the evolving therapeutic options for patients with these conditions.

A “take-home” message from this study is that in cases of pressure-coupled (i.e., pressure-dependent) lesions of the thalamus and ventricles, clinical severity likely results from an interplay among the geometry of the lesion, the reserve of the networks affected by the lesion, and the intracranial compliance; thus, clinical severity cannot be solely assessed based on the volume of the lesion alone. Therefore, a useful, reproducible method for addressing this problem is through pairing preoperative mechanism-based stress phenotyping with a logical, sequential decompression protocol that addresses compartmental relaxation first, and then employs explicit stopping rules based upon the amount of functional tissue containing perforating vessels encountered during surgery. This approach should allow for more informed and conservative decisions regarding surgery, while providing a means of assessing the impact of surgery longitudinally using objective endpoints that will continue to have relevance as the molecular characteristics of tumors are increasingly used to guide post-surgical treatment strategies.

## Figures and Tables

**Table 1:** Selected contemporary studies informing the interpretation and management of deep thalamic and intraventricular lesions. The table summarizes original research and consensus-level data addressing network-level clinical phenotypes, ventricular compliance and periventricular microenvironmental biology, operative strategy in perforator-dense deep corridors, and molecular or biomarker-driven therapeutic implications.

References	Study Type	Population	Key Findings	Direct Relevance to Current Case
[20,21]	Consensus classification	CNS tumors (adult + pediatric)	Defines integrated entities using histology + molecular alterations ± methylation class; midline location mandates active molecular adjudication (e.g., H3 K27–altered DMG vs. IDH-wt GBM-spectrum mimics).	Justifies why a thalamic epicenter with heterogeneous enhancement cannot be biologically presumed; supports your “tissue + class assignment” logic in a boundary-limited corridor.
[22]	Guideline	Adult diffuse gliomas	Frames care as maximal safe cytoreduction when feasible + RT ± TMZ stratified by biomarkers (MGMT, IDH, H3 status); deep/eloquent lesions emphasize function-first surgical intent.	Aligns your operative philosophy (decompression + representative tissue + boundary respect) with contemporary “safe maximalism,” not margin pursuit.
[23]	Trial/practice-defining evidence	High-grade glioma cohorts	Establishes RT–TMZ as backbone for eligible malignant glioma; supports early surgical steps as gateway to adjuvant disease control.	Supports your framing that surgery served dual goals: pressure–network uncoupling + diagnostic entry into biomarker-stratified therapy.
[24]	Biomarker outcome studies	GBM treated with TMZ	MGMT methylation stratifies alkylator benefit and prognosis; reinforces the need for standardized biomarker reporting when the extent of resection is constrained.	Makes biomarker acquisition clinically “non-optional” in deep thalamic tumors where resection is intentionally boundary-limited.
[25]	Retrospective series/reviews	Adult thalamic gliomas ± ventricular involvement	Adult thalamic tumors often present with cognitive/behavioral decline and non-motor disability; resection extent is limited by perforators/fiber compaction; survival and morbidity depend on biology + safe decompression.	Provides context that your case is typical in risk geometry yet distinctive in high-resolution phenotyping and physiology-first framing.
[26]	Systems/cognitive neurology	Thalamic/perithalamic lesions	Deep lesions can preferentially impair processing speed, interference control, set-shifting, language retrieval, and dual-task integration with minimal paresis early; deficits track hub/gating disruption.	Directly supports your quantified phenotype (Stroop/TMT cost, SDMT, fluency, dual-task TUG) as mechanistically localizing, not descriptive.
[27]	Clinico-radiologic/hydrocephalus-adjacent literature	Ventricular-adjacent masses	Posterior ventricular distortion can behave as a compliance amplifier: nausea/vomiting and fatigue-amplified collapse may track compartment stress and CSF buffering failure, not edema alone.	Supports your central concept: intraventricular extension is not incidental; it is the bridge between network collapse and pressure physiology.
[28]	Surgical technical series	Intraventricular + deep midline lesions	“Sequence-driven” safety: graded CSF release, inside-out debulking to reduce traction vectors, source-directed hemostasis, minimal thermal spread, strict respect for ependymal/subependymal veins and perforator interfaces.	Maps onto your operative narrative and explains why the technical novelty is physiologic stability during geometric change, not exposure theatrics.
[29]	Translational + clinical feasibility	Glioma, enriched for deep/midline	CSF often outperforms plasma for tumor signal; supports molecular ascertainment when tissue is limited; enables longitudinal monitoring of residual biology.	Particularly relevant in thalamic–ventricular lesions where sampling bias is plausible; future-proofs your discussion with practical biomarker pathways.
[30]	Translational + emerging clinical data	DMG cohorts incl. thalamic	Highlights that correct class assignment can route patients to mutation-directed strategies and trials; underscores that “glioblastoma-like” imaging can mask distinct biology.	Strengthens your argument that molecular classification has actionability consequences in exactly this location/behavior pattern.

## Data Availability

The data presented in this study are available on request from the corresponding author.
